# Preliminary evidence of extrarenal sodium storage in a large mammal: implications for comparative physiology and hypertension research: Running: Sodium storage in cattle

**DOI:** 10.1007/s00424-026-03155-2

**Published:** 2026-02-28

**Authors:** Andrew J. Abraham, Ethan S. Duvall, Callum Leese, Kirstin Abraham, Elizabeth le Roux, Barbara Riond, Sylvia Ortmann, Melissa Terranova, Graham Leese, Matthew A. Bailey, Marcus Clauss

**Affiliations:** 1 Center for Ecological Dynamics in a Novel Biosphere (ECONOVO), Section of EcoInformatics and Biodiversity, Department of Biology, Aarhus University, Aarhus, Denmark; 2 School of Informatics, Computing and Cyber Systems, Northern Arizona University, Flagstaff, USA; 3 Department of Ecology and Evolutionary Biology, Cornell University, Ithaca, USA; 4 Department of Population Health and Genomics, Ninewells Hospital, University of Dundee, James Arnott Drive, Dundee, UK; 5 University of Dundee Medical School, Ninewells Hospital, James Arnott Drive, Dundee, UK; 6 Mammal Research Institute, Department of Zoology and Entomology, University of Pretoria, Pretoria, South Africa; 7 Clinical Laboratory, Department for Clinical Diagnosis and Services, Vetsuisse Faculty, University of Zurich, Winterthurerstr. 260, Zurich, 8057 Switzerland; 8 Leibniz-Institute for Zoo and Wildlife Research (IZW), Alfred-Kowalke-Str. 17, Berlin, 10315 Germany; 9 AgroVet-Strickhof, ETH Zuirch, Lindau, 8315 Switzerland; 10 The Centre for Cardiovascular Science, The Institute of Neuroscience and Cardiovascular Research BioQuarter Campus, The University of Edinburgh, Edinburgh, EH16 4TJ UK; 11 Exotic Pets and Wildlife, Vetsuisse Faculty, Clinic for Zoo Animals, University of Zurich, Winterthurerstr. 260, Zurich, 8057 Switzerland; 12 AgroVet-Strickhof, Vetsuisse Faculty, University of Zurich, Lindau, Switzerland

**Keywords:** Allometry, Cattle, Homeostasis, Hypertension, Potassium, Renal, Sodium

## Abstract

Under conditions of dietary sodium (Na^+^) excess, the kidneys may fail to adequately excrete Na^+^, potentially compromising blood pressure homeostasis. Body tissues, such as skin, can offer sites of short-term extrarenal Na^+^ storage and previous research has shown that this can help guard against hypertension in small mammals (e.g., rodents). Large mammals have relatively greater Na^+^ storage potential, but whether extrarenal Na^+^ storage occurs for this group is unknown. Here, we report preliminary evidence of extrarenal Na^+^ storage in cattle. We provided a large pulse-dose of NaCl to four cattle (body mass: ~720 kg) and measured excretion of Na^+^ and potassium (K^+^) in urine and faeces for a period of 7-days. Following NaCl administration, Na^+^ excretion spiked in both urine and faeces for ~ 48 h before returning to baseline measurements. After ~ 96 h, however, Na^+^ excretion increased again; a consistent physiological phenomenon across all individuals studied. We did not observe a pattern in urinary K^+^ excretion, indicating that the mechanism of Na^+^ storage does not appear to involve exchange for K^+^. However, faecal K^+^ excretion was reciprocal to that of Na^+^, presumably reflecting exchange of Na^+^/K^+^ across the walls of the large intestine. We infer that during the initial period of Na^+^ stress, short-term extrarenal Na^+^ storage occurred and the stored Na^+^ was later released only when the body had returned to Na^+^ homeostasis. Additional experiments are required to understand how patterns of Na^+^ regulation changes across body sizes and the specific body compartments involved. Cattle may be a useful model system for examining the impact of high Na^+^ intake in mammals larger than humans.

## Introduction

Understanding how mammals, including humans, regulate excess sodium (Na^+^) from their bodies remains a critical research prioirity [1]. Na^+^ is essential for a variety of homeostatic, metabolic, neural, and muscular processes; however, short-term imbalances in Na^+^ directly alter extracellular fluid (ECF) volume and blood pressure [2]. In cases where excess Na^+^ intake occurs chronically, hypertension may develop, leading to various cardiovascular diseases and premature death [3]. Indeed, in Western countries, where Na^+^ is commonly consumed in excess, hypertension accounts for 18% of all cardiovascular diseases in humans, including kidney failure, strokes, and coronary heart disease [4]. In 2010, it was estimated that 31.1% of the global adult population (1.39 billion people) had hypertension [5]. Still, there remains debate about the exact mechanisms of Na^+^ regulation in the mammallian body [1, 6, 7], including the role of body size [8] and individual salt sensitivity [6].

Mammals assimilate the vast majority (> 90%) of the Na^+^ they consume into their bloodstream [9, 10]. Yet, despite wide variation in total Na intake, adaptive neural and hormonal responses typically act to maintain blood plasma Na^+^ within the narrow limits required to maintain osmotic balance and blood pressure [11]. The kidneys are the main regulators of Na^+^ balance, removing excess Na^+^ from the bloodstream and excreting it in urine [9, 10]. Additionally, excess Na^+^ within the bloodstream may be resecreted into the large intestine for elimination in faeces, although this pathway is typically an order of magnitude smaller than urinary excretion [12]. However, when Na^+^ intake exceeds the capacity of the renal and digestive systems to rapidly achieve Na+ balance [13], the body can evoke a third mechanism: extrarenal storage of Na^+^ ions in body tissues [14, 15].

Conventionally, it has been considered that Na^+^ can be stored in two major compartments of the body: one circulating the body in plasma and one slowly exchangeable in bone [1, 11]. However, recent research has highlighted another storage compartment within the interstitium [11, 13]. In the interstitial spaces of vascular endothelium, skin, and muscle, surplus Na^+^ from the bloodstream can bind to negatively charged glycosaminoglycans (GAGs), acting as a potential reservoir for when the body is stressed by overdoses of Na^+^ [13]. Importantly, however, it appears that the body can store this surplus Na^+^ in the interstitium without proportional increases in water or losses of potassium (K^+^), which are typically required to maintain extracellular osmolarity via the sodium-potassium pump (Na^+^/K^+^-ATPase) [13, 16]. Nevertheless, this mechanism remains poorly understood and is at the center of debate regarding whether Na^+^ bound to GAGs is ostomically inactive or simply relects extracellular volume expansion [17]. An alternative explanation posits that Na^+^ can accumulate intracellularly through exchange for K^+^ [18], which should subsequently be reflected in increased urinary K^+^ excretion during periods of extrarenal Na^+^ storage. Yet, evidence for either osmotically active or inactive Na^+^ storage is equivocal [9, 13, 19], in part due to the influence of multiple confounding factors on electrolyte excretion rates including age, circadian rhythms, and individual salt sensitivity [6, 20]. New models for the distribution and dynamics of osmotically inactive Na^+^ are required to advance our understanding of the underlying mechanisms governing Na^+^ homeostasis [9].

We recently proposed that the requirement and capacity of mammals to envoke extrarenal Na^+^ storage may be related to body mass (BM) [8]. Our reasoning follows the logic that, while the rate of Na^+^ excretion in urine and faeces is governed by metabolic processes that scale hypoallometrically at ~BM^0.75^, the relative mass of skin (BM^0.97 (95% CI: 0.96–0.98)^ [21]), muscle (BM^1.01 (95% CI: 0.99–1.03)^ [22]), and bone (BM^1.10 (95% CI: 1.08–1.12)^ [23]), scale isometrically or hyperallometrically. These scaling differences suggest that larger-sized mammals may possess a higher capacity for extrarenal Na^+^ storage compared to their smaller counterparts. Accordingly, evaluating whether large mammals can envoke extrarenal Na^+^ storage and how interrelated electrolytes (e.g., K^+^) respond may hold novel insights for understanding this poorly understood mechanism, with potentially informative application for human health. To date, however, most experimental research on extrarenal Na^+^ regulation has focused on humans and rats, with an individual study each on pigs and dogs [16, 24–28].

Here, we present preliminary evidence that a large mammal (cattle) displays short-term extrarenal Na^+^ storage in body tissues without altering K^+^ excretion rates. During the course of another study investigating Na^+^ passage through the bodies of large mammals [12], our data revealed a pattern corresponding to apparent Na^+^ storage in body tissues. We describe this serendipitous result and discuss why large mammals, and specifically domesticated cattle, may be a useful study system for understanding the mechanisms of Na^+^ storage and release across body sizes. Finally, we describe the relevance of these results for future research on Na^+^ regulation and hypertension in mammals, including humans.

## Methods

This experiment was conducted at the AgroVet-Strickhof research facility, Switzerland, during February 2024, under the experimental licence 35,775|ZH059/2023.

### Study animals and husbandry

We used four rumen-fistulated Original Brown Swiss cows (body mass range: 700–740 kg) in the final stage of lactation (milk yield 6–8 L/d). Animals were part of the AgroVet-Strickhof research herd living in a free stall, and had received rumen fistulae several years prior to the present study. They were brought to the tie stall (to which they were habituated from other previous experiments) three days prior to the experiment. Each cow was tethered in an individual stall with chopped straw bedding and *ad libitum* access to water. Water consumption was electronically recorded every 15 min in units of 1000 ml. Cows were kept on total mixed ration based on grass and maize silage for the duration of the experiment, which had a Na concentration of 5185 mg kg^− 1^ dry matter. Each morning, 30 kg of fresh total mixed ration was provided onto a feeding platform, with an additional 20 kg supplied in the evening. At the beginning of each day, all remaining food was collected and weighed. As there were always leftovers, this was considered feeding for *ad libitum* consumption. One day prior to the experiment, cows were fitted with urinals custom-made from neoprene diving suits attached around the vulva of the cows and fixed by hook-and-loop fastener straps glued (Ergo 5011; Kisling AG, Wetzikon, Switzerland) onto the skin. The urinals were connected through a pipe to a canister on the ground for urine collection. The rear end of the stand was formed by a grid through which defecated faeces fell into a waste canal; sufficient material remained on these grids at each defecation for sampling, after which they were cleaned to avoid contamination of the subsequent sample.

### Sodium pulse experiment

We first collected control urine and faeces from all animals (*n* = 2–3 per individual) on the day prior to the Na dosage treatment. Samples of urine were taken from the canister, and samples of faeces were collected from the defecation grid. For each animal, we then administered a pulse dose of salt (NaCl; 400 g per cow). We used a large pulse of NaCl to maximise the opportunity of observing a signal in Na^+^ retention times across both urinary and faecal pathways [12]. However, this protocol also created circumstances under which extra-renal Na^+^ storage may occur [13]. We ensured to administer within safe Na limits. According to veterinary sources (CliniTox https://www.vetpharm.uzh.ch/clinitox/toxdb/WDK_072.htm and [29]), clinically dangerous doses in animals with *ad libitum* water access would have been 1.2 kg of salt for cows. Salt was manually inserted into the top particle layer of the rumen contents via the fistula.

We collected urine and faeces for each individual over seven days (168 h) at progressively longer intervals (interval on days 1–2 = 4 h; days 3–4 = 6 h; day 5 = 8 h; days 6–7 = 12 h; total *n* = 26 per individual). The defecation grids were checked at least every four hours to collect faeces, even when the respective sampling interval was longer. In this setup, urine and faecal samples were available for all animals at all sampling intervals. At the end of each interval, urine collected in the canisters was weighed prior to sampling and then ~ 150 ml extracted via pipette. All urine samples were immediately frozen at −20 °C. The urine canister was then cleaned and replaced between each sampling interval. Faeces collected during a collection period were mixed homogenously, and a representative sample of ~ 250 g fresh material was taken. Faeces were then dried at 105 °C for 72 h and ground in a feed mill (Schmersal GmbH, Wuppertal, Germany) using a 1 mm matrix.

### Sample analysis

We measured the concentration of Na^+^, K^+^, and creatinine in urine. Urine element concentrations are susceptible to dilution; for example, in response to the high salt dose, animals may increase drinking water intake and thus their urine volume [30]. Creatinine can be used to correct for this dilution effect as the daily excretion of urinary creatinine is considered constant, and hence deviations in its concentration are an indication of the degree of dilution [31, 32]. Na^+^ and K^+^ in urine were measured using indirect ISE (ion-selective-electrode) and creatinine was determined using the Jaffé-method. Both analytes were measured using a fully automated chemistry analyzer (Cobas C 501, Roche Diagnostics, Switzerland). For faeces, we used inductively coupled plasma optical emission spectrometry (ICP-OES, model Optima 8000, Perkin Elmer) after wet ashing [33].

### Total sodium and potassium excretion rates

To compare Na^+^ and K^+^ excretion via urine and faecal pathways, we quantified total losses by each pathway. For urine, we simply multiplied the total urine mass by elemental concentrations in each sampling period. For faeces, we did not measure dry matter output per sampling period; however, we did record daily dry matter intake (DMI) and can make a reasonable assumption on the digestibility of this particular diet based on a meta-analysis in cattle [34]. We could therefore make reasonably accurate estimates of faecal dry matter rates by using Eq. 1:

1 $$\:F_i=\frac{{DMI}_{i-2}\:\ast\:A\:}T$$

Where, F_i_ is faecal dry matter production in kg DMI/hr during sampling period *i*, DMI_i−2_ is the dry matter intake two days prior based on a gut passage time of 41–54 h in cows [12], A is a dry matter assimilation rate of 77.7% [34] and T is the duration of sampling period *i* in hours. As with urine, to calculate total faecal Na^+^ and K^+^ excretion via the faecal pathway, we multiplied our estimates of faecal dry matter production with elemental concentration for each sampling period. As our intervals became progressively longer, we standardised all results per hour.

### Statistical analysis

Although our sample size was small (*n* = 4 cows), the high number of samples collected per individual allowed us to statistically examine if the excretion rate of Na^+^ and K^+^ altered through time (total *n* = 104). All statistical procedures were undertaken in R statistical software v4.3.1. First, we undertook repeated measures analysis of variance (rmANOVA) using the ‘afex’ packageand included individual cow as a random effect. We applied this for excretion rates of Na^+^ and K^+^ in urine and faeces individually, whereby p-values < 0.05 would indicate that the rate of elemental excretion statistically changed during the Na-loading experiment. However, rmANOVA assumes linear trends, whereas visual inspection of our data revealed non-linear effects. Following Mundo et al. [35], we therefore also fitted generalised additive mixed models (GAMMs) using the ‘mgcv’ package. GAMMs fit smooth, flexible functions or splines to the data allowing for potentially non-linear patterns [36]. Specifically, we fit splines with time as the predictor variable and again included individual cow as a random effect for each element individually. A high marginal R^2^ (variance explained by fixed effects) and a p-value < 0.05 would indicate that the model can statistically explain rates of elemental excretion through time. All models were checked using gam.check() and were determined to be sufficiently robust based on normality of residuals and homoscedasticity.

## Results

### Na^+^ administration

The mean food intake per cow was 16.3 (SD = 2.0) kg DM/day, representing a daily background Na^+^ intake of 84.5 ± 10.4 g per animal. At t = 0, we administered a 400 g dose of NaCl, equivalent to an additional 156 g of Na^+^ intake.

### Phase 1: Filtration of excess Na^+^ by kidneys and short-term extrarenal Na^+^ storage

**Fig. 1:**
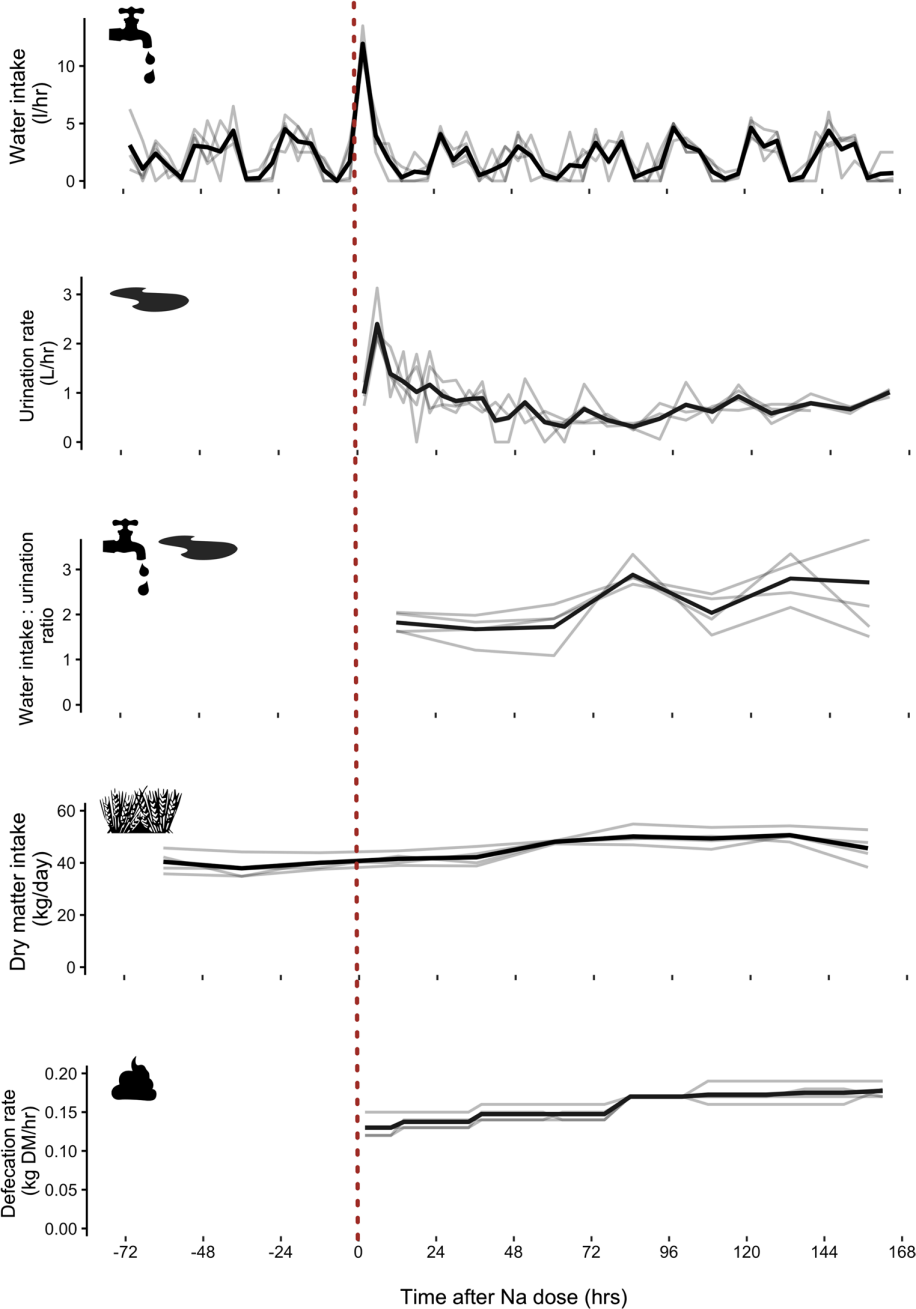
Rates of water intake, urinary output, water intake : urinary output ratio, dry matter intake and faecal output by four cattle measured over a ten-day period. Black and grey lines represent mean and individual values, respectively. The dashed red line represents when a 400 g pulse dosage of salt (NaCl) was administered to cattle

**Fig. 2:**
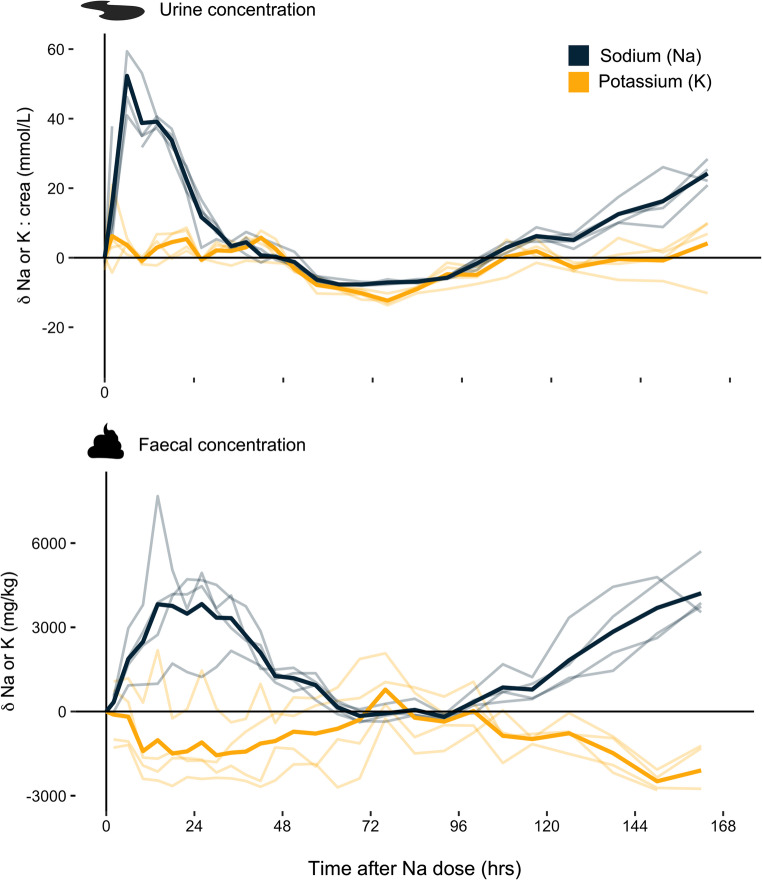
Change in the concentration of sodium (Na^+^) and potassium (K^+^) in urine and faeces over seven days following the administration of a large 400 g pulse dose of NaCl at t = 0. In urine, concentrations are reported as ratios relative to creatinine (crea) to correct for possible dilution effects due to changing urine volume. All ratios/concentrations are reported relative to baseline values measured on the day prior to salt administration. Thick and thin lines represent mean and individual values, respectively

**Fig. 3:**
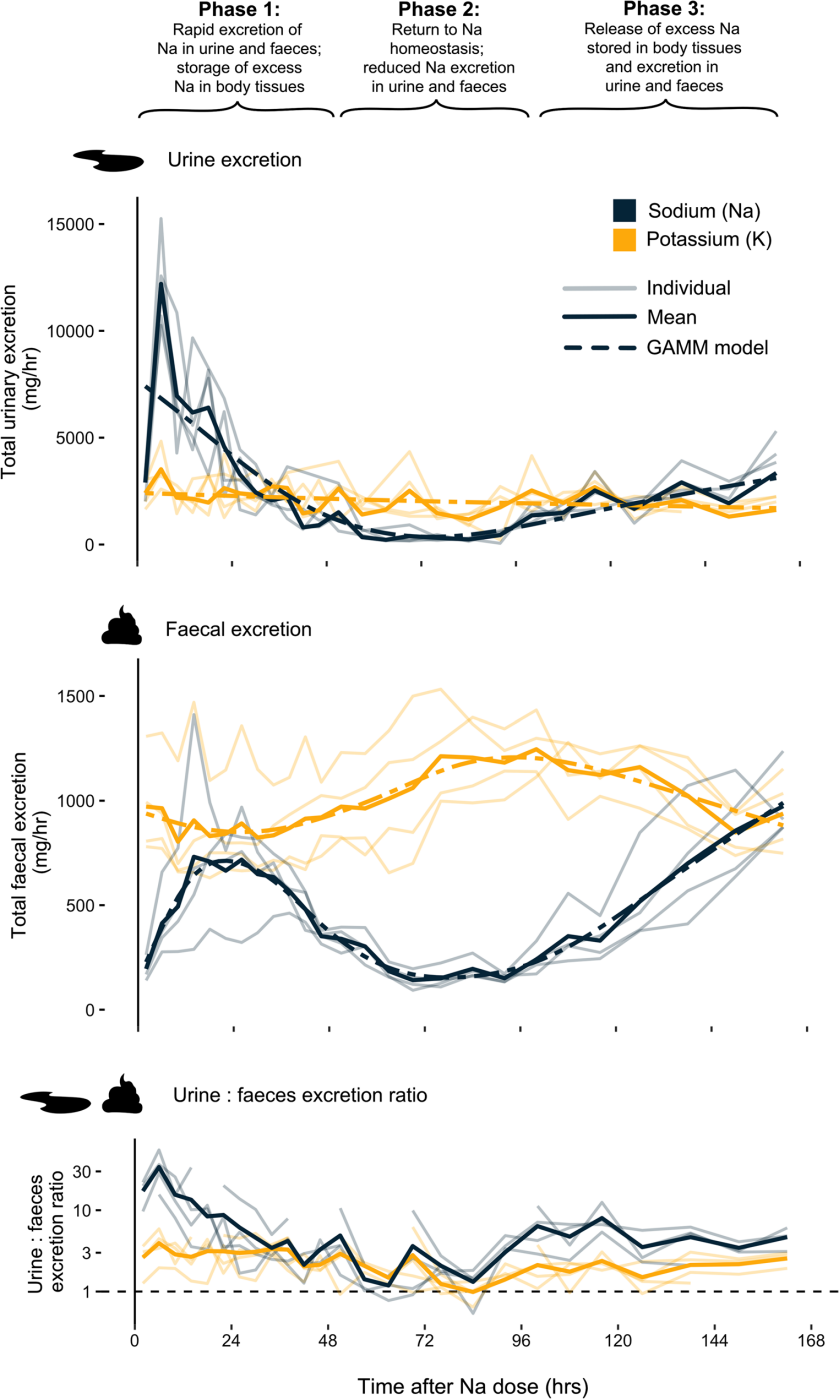
Sodium (Na^+^) and potassium (K^+^) excretion patterns observed in urine and faeces over seven days after a large pulse dose of salt (400 g of NaCl) was administered to four cattle (*Bos taurus*). Three distinct phases of Na^+^ regulation are highlighted and correspond close for both urinary and faecal excretion pathways. Note the difference in magnitude denoted by high urine: faecal ratios, which is presented on a log_10_ axis (bottom panel)

Following the administration of NaCl, all cows immediately increased water consumption to > 10 L/hr compared to a daily maximum of < 5 L/hr in the three preceding days (Fig. 1a). This corresponded to a total water consumption of 78 (SD = 7) L in the 24 h after NaCl dosage compared to a baseline daily water intake of 49 (SD = 6) L/day. As a result, urine output also spiked to 2.4 (SD = 0.5) L/hr in the period 4–8 h after NaCl administration, following which urine rates gradually decreased to ~ 0.5 L/hr over the next two to three days (Fig. 1b). The drinking water intake: urination ratio was comparatively low at this stage (~ 1.8), suggesting that the majority of water intake was used for renal excretion (Fig. 1c). Urine Na^+^ concentrations spiked quickly, with highest Na: creatinine ratios occurring 4–8 h after salt dosage and returning to baseline levels after 48 h (Fig. 2). Together, the dramatic increase in urine volume and Na^+^ concentration resulted in total urinary excretion exceeding 12,000 mg/hr, before gradually returning to baseline levels of < 1,000 mg/hr. Urinary K^+^, on the other hand, did not show any response to salt administration (Fig. 3). Faeces displayed a slower response than urine and Na^+^ concentrations peaked at 26 h after salt administration, with a more symmetrical pattern of increase and decrease through to 72 h. Faecal K^+^ displayed a mirror inverse pattern to faecal Na^+^, decreasing for 24 h before increasing to baseline levels (Fig. 2). The maximum rate of Na^+^ excretion in faeces during phase 1 reached ~ 700 mg/hr; an order of magnitude less than urine.

### Phase 2: Return to Na homeostasis

In both urine and faeces, total Na^+^ excretion returned to baseline levels between 48 and 96 h (urine; 200–500 mg/hr) and 72–96 h (faeces; 150–200 mg/hr) after NaCl administration (Fig. 3). At this stage, the magnitude of Na^+^ and K^+^ excretion was comparable through both excretion pathways and represented just ~ 3% and ~ 20% of the maximum Na^+^ excretion rates observed in phase 1 for urine and faeces, respectively (Fig. 3). During phase 2, both urinary Na^+^ and K^+^ concentrations dipped consistently below baseline levels, whereas faeces returned to almost exactly the same pre-treatment concentrations (Fig. 2).

### Phase 3: Gradual release of stored extrarenal Na

After 96 h, Na^+^ concentration began linearly increasing in both urine and faeces (Fig. 2). Urine K^+^ remained at baseline levels, but faecal K^+^ again displayed a mirror inverse of faecal Na^+^ and began decreasing. The observed increase in Na^+^ excretion was also mirrored by a slight increase in water intake and urine production, but the ratio of water intake: urine increased (> 2), together with food intake and faecal excretion (Fig. 1). On day 7, at the termination of the experiment, total urine Na^+^ excretion was ~ 4,500 mg/hr; only ~ 35% of the maximum peak observed in phase 1, but > 10 times higher than Na^+^ excretion observed during phase 2. Faecal Na^+^ excretion, on the other hand, exceeded the phase 1 peak at > 950 mg/hr and was > 5 times higher than faecal Na^+^ excretion observed during phase 2 (Fig. 3). During the second peak in Na^+^ excretion, the urinary pathway was 3–5 times greater than the faecal pathway.

### Statistical models of elemental excretion rates through time

All rmANOVA models were statistically significant at *p* < 0.05 (Table 1), indicating that excretion rates statistically altered through time. Notably, the effects sizes (η²) were larger for Na^+^ than K^+^, and the p-value for K^+^ in urine was marginal (*p* = 0.042). GAMMs fitted to elemental excretion rates produced high marginal R^2^ > 0.60 for Na^+^ with time a significant predictor in all cases (*p* < 0.001) (Table 2). By contrast, model fits were poorer for K^+^ with fixed effects explaining only 10% and 29% in urine and faeces, respectively.

**Table 1:** Results of repeated measures analysis of variance (rmANOVA) for models fitted to the excretion rate of sodium (Na^+^) and potassium (K^+^) through time in urine and faeces with individual cattle included as random effects. Note: during some time intervals, some individual cattle didn’t produce urine samples reducing overall sample size for this measurement. All models were significant (*p* < 0.05) indicating that excretion rates changed during the Na-loading experiment; larger effect sizes denote the magnitude of change

Sample	Element	df (effect)	df (error)	F-value	*p*-value	Effect size (η²)
Urine	Na	18	54	23.449	< 0.001	0.869
	K	18	54	1.848	0.042	0.354
Faeces	Na	25	75	12.117	< 0.001	0.696
	K	25	75	4.650	< 0.001	0.321

**Table 2:** Results of generalised additive mixed models (GAMMs) fitted to the excretion rate of sodium (Na^+^) and potassium (K^+^) through time in urine and faeces with individual cattle included as random effects. Time is significant (*p* < 0.05) in all models, but variably explains element excretion through time. R^2^ (conditional) represents total model fit and R^2^ (marginal) represents that exaplained by the fixed effects (i.e., time) only. For plots of GAMM models, see Fig. 3

Sample	Element	Term	edf	F-value	*p*-value	*R* ^2^ (conditional)	*R* ^2^ (marginal)
Urine	Na	S(Time)	4.589	24.56	< 0.001	0.616	0.608
	K	S(Time)	1.897	4.34	0.012	0.157	0.101
Faeces	Na	S(Time)	7.477	38.36	< 0.001	0.810	0.679
	K	S(Time)	5.255	17.37	< 0.001	0.762	0.291

## Discussion

### A plausible explanation for delayed Na^+^ release: short-term extrarenal Na^+^ storage

As the findings in this paper were serendipitiously observed during the course of another experiement [12], we did not collect all measurements necessary to definitively resolve the physiological mechanisms behind our observations. Nonetheless, the patterns described offer a strong basis for informed speculation on what may have occurred. We discuss potential explanations for apparent extrarenal Na^+^ storage, as well as how these finding can help guide future research below. In due course, follow-up studies may confirm the accuracy of these speculations.

**Fig. 4:**
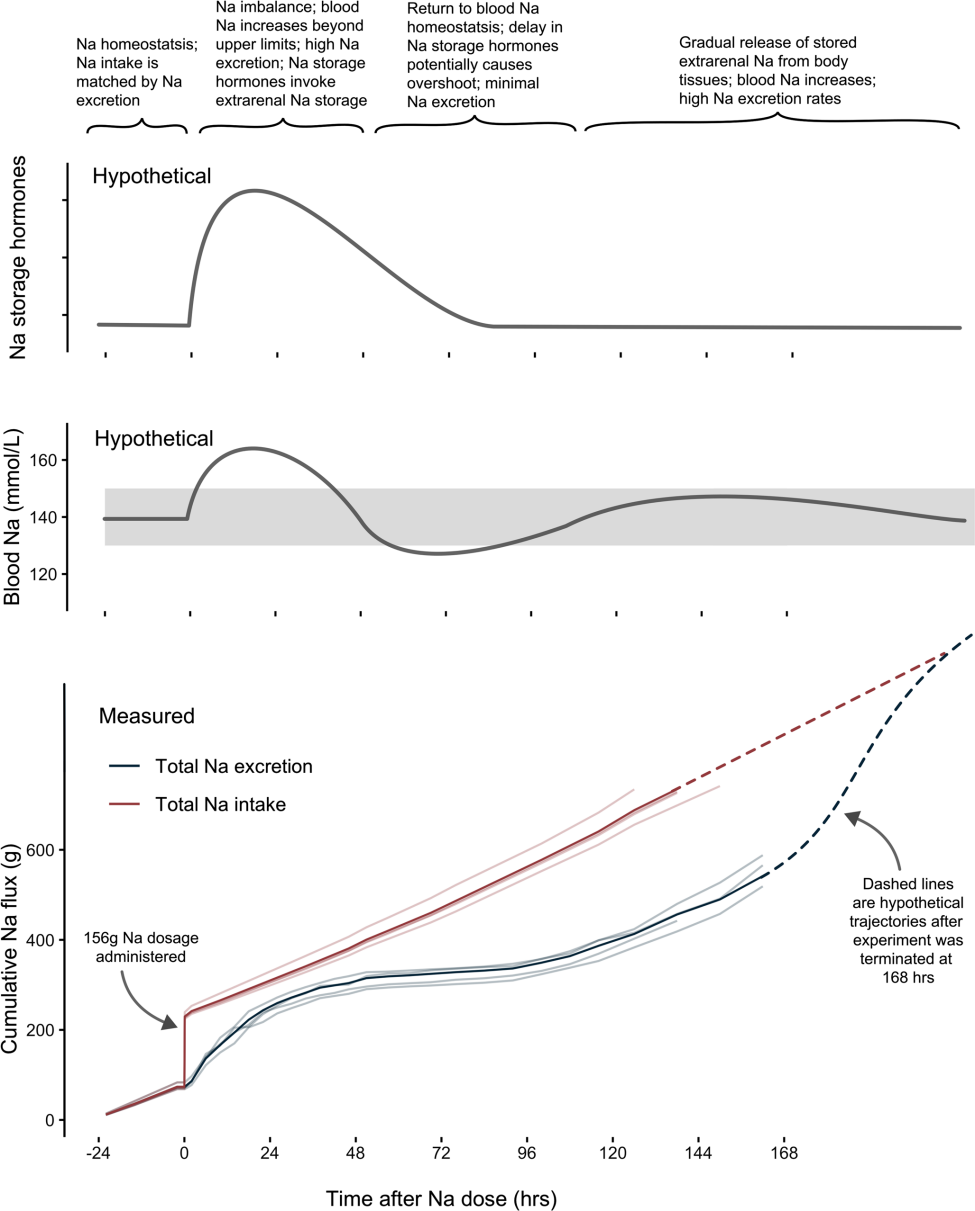
Cumulative total intake and excretion of sodium (Na^+^) over seven days after a large pulse dose of salt (400 g of NaCl) was administered to four cattle (*Bos taurus*). Prior to the experiment, cattle were in Na^+^ homeostasis, with Na excretion equal to intake. Following Na^+^ dosage, the intake (red) line goes above the excretion (blue) line, indicating a net increase of Na^+^ in the body. Only once these lines intersect again will all additional Na^+^ have been released. Interestingly, Na^+^ excretion levels off prior to this occurring between 48–96 h. One possible explanation is that there was a mismatch between hormones controlling Na^+^ excretion and extrarenal Na^+^ storage in body tissues as depicted by the hypothetical graphs above. In this instance, a strong surge of hormones driving extrarenal Na^+^ storage in the period 0–72 h may have caused low blood Na^+^ concentrations. It is only after this hormonal drive that blood Na^+^ can return to normal and Na^+^ stored in body tissues be rereleased (96–168 h). Unfortunately, this experiment was terminated before all additional Na^+^ from the experiment had been excreted. Additional experiments should determine if the depicted hypothetical scenarios of blood and Na^+^ storage hormones actually occurs

Prior to the Na^+^ loading experiment, cattle were assumed to be in Na^+^ homeostasis, with Na^+^ excretion equal to Na^+^ intake (Fig. 4). Following the administration of a very large pulse Na^+^ dosage, we infer the following: Na^+^ was (presumably) quickly assimilated into the bloodstream, and exceeded the narrow limits required to maintain osmotic balance and blood pressure [11]. This likely resulted in a hormonal response to (i) drink more water, (ii) excrete surplus Na^+^ in urine, and (iii) invoke extrarenal Na^+^ storage (Fig. 4), and (iv) exchange Na^+^ for K^+^ in the large intestine and excrete additional Na^+^ in faeces (Fig. 2). Indeed, although our sample size is small, all individuals displayed remarkably consistent physiological responses that strongly indicated increased Na^+^ excretion and temporary storage of excess Na^+^—mechanisms that serve to buffer blood Na^+^ levels and avoid adverse health impacts following overconsumption of Na^+^, including hypertension [2].

After 48 h, it appears that blood Na^+^ concentrations may have stabilised, inferred from Na^+^ excretion rates (Fig. 3). Indeed, there may have even been an overshooting storage reaction with low blood Na^+^ concentrations resulting in the observed very low excretion rates of Na^+^ between 48 and 96 h after salt administration (Figs. 2 and 3). It is possible that this overshoot occurred due to a delay in hormonal mechanisms monitoring concentrations of Na^+^ in blood and those driving the storage or excretion of surplus extrarenal Na^+^ (Fig. 4). After 96 h, however, it appears that the cattle had recovered from the physiological aftershock of being supplied with a very large dose of NaCl. At this point, we suspect that Na^+^ stored in body tissues re-entered the bloodstream and was excreted primarily in urine, with second order losses from faeces (Figs. 3 and 4).

Unfortunately, our experiment was terminated before the cattle had once again reached Na^+^ homeostasis. As such, we are unsure of how long it took for the cattle to release all of the Na^+^ administered at t = 0. Similarly, it is possible that electrolyte concentrations observed in urine and faeces were also reflected in milk (cattle produced 6–8 L/day); however, we did not measure this matrix in our original study [12], although we do not believe that Na losses via milk would have substantially altered our conclusions of apparent extrarenal Na^+^ storage inferred from excretion rates in urine and faeces.

### Inferred mechanism of extrarenal Na^+^ storage

**Fig. 5:**
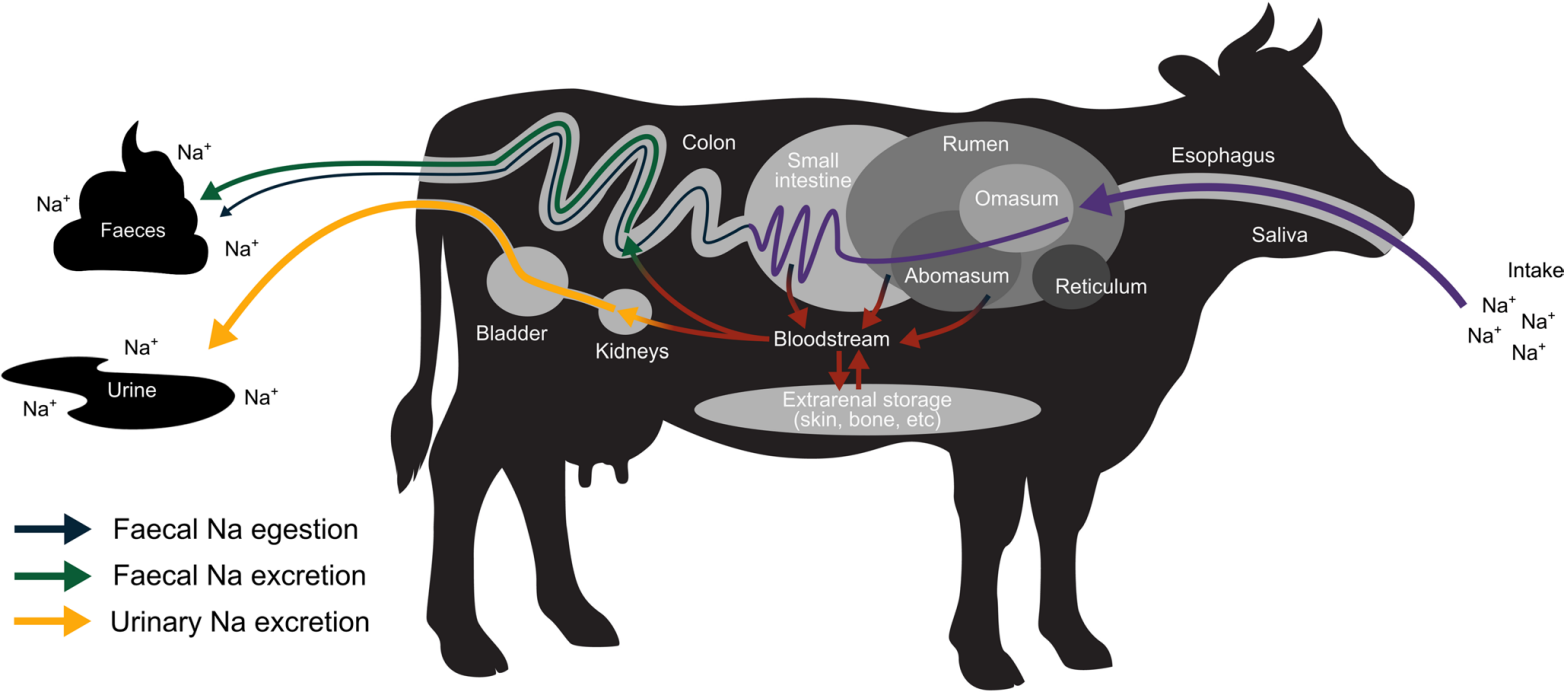
Sodium (Na^+^) regulation in cattle (*Bos taurus*) modified from Abraham et al. (2025). Ingested Na^+^ is mostly absorbed into the bloodstream in the upper gastrointestinal tract (rumen – small intestine). Under conditions of high Na^+^ intake, the kidneys cannot filter sufficient Na^+^ from the blood to prevent hypertension. Accordingly, surplus Na^+^ is stored in extrarenal body tissues (e.g., skin, muscle, bone) for later release via urinary and faecal excretion pathways. Large mammals theoretically have greater extrarenal Na^+^ storage capacity than smaller animals, such as rats; however, little is known about the mechanisms involved. With their ease of handling and management, cattle might represent attractive model organisms to explore such questions further

We did not directly measure Na^+^ storage in body tissues [12], but we interpret the consistent pattern of a second peak in Na^+^ release across individuals as being strongly suggestive of delayed mobilization of excess Na^+^ stored during the initial period of Na^+^ overload [15] (Table 2; Fig. 3). This hypothesis is consistent with existing evidence of extrarenal Na^+^ storage in rats, pigs, and humans [16, 24–28] (Fig. 5).

Interestingly, we found that K^+^ excretion in urine remained consistent throughout our experiment with cattle (Fig. 3); the effect significance in the rmANOVA model was marginal (*p* = 0.042; Table 1) and the GAMM model only explained 10% in fixed effects (Table 2). This result indicates that K^+^ is not excessively released into the bloodstream when Na^+^ is transported into body tissues for osmotically inactive storage. This observation stands in contrast to theories that suggest extrarenal Na^+^ storage is dependent on exchange with intracellular K^+^ [9, 18, 19]. Even more, we found that faecal K^+^ displayed an inverse pattern to faecal Na^+^ at the time of presumed extrarenal Na^+^ storage (Fig. 2), most likely reflecting an exchange of K^+^ when Na^+^ is transferred from the bloodstream across the walls of the large intestine, as describe earlier in cattle [37]. Nevertheless, this retention of K^+^ was not large enough to be reflected in urinary concentrations. Because the magnitude Na^+^ and K^+^ release via the faecal pathway is small compared to urinary losses (up to 30x smaller during peak Na^+^ excretion periods; Fig. 3), the associated change to total body K^+^ flux over the course of our seven day experiment is small. Ultimately, this finding confirms that Na^+^/K^+^ exchange processes linked to short-term Na^+^ balance can only generate detectable patterns in a faecal K^+^ response—patterns that were absent in urine.

Due to the lack of prominant Na^+^/K^+^ exchange in urine, combined with the short timescale of Na⁺ storage and release, we speculate that excess Na^+^ ions in our study may have bound to GAGs within the interstital spaces of skin and muscle [11, 13]. GAGs are highly negatively charged polysaccharides found abundantly in the extracellular matrix of soft tissues such as skin and muscle. Because binding of Na^+^ to GAGs occurs extracellularly and does not involve cellular uptake, there is no immediate need for a compensatory K⁺ exchange to maintain intracellular membrane potential. While bone could also serve as a Na⁺ storage site without requiring direct K⁺ exchange on and within the bone mineral crystal surface [28, 38], the rapid storage responses we observed likely exceeded the speed expected for most bone-mediated exchange, which generally occurs over longer timescales [38]. In contrast, the reversible binding of Na⁺ to GAGs can occur rapidly, enabling the body to adjust Na⁺ storage dynamically in response to short-term fluctuations in intake or plasma levels [11, 13]. We did not have measurements for other ions (e.g., Ca^+^, Mg^+^, Cl^−^) or any blood measurements, which might have added further insights into the processes involved. In particular, Cl^−^ may hold useful insights as its presence may influence to what degree the storage process is osmotically relevant [30, 39].

### Why bigger may be better when studying mechanisms of extrarenal Na^+^ storage in mammals

We believe that our results are the first time that evidence for extrarenal Na^+^ storage has been suggested in a mammal species larger than humans. This aligns with theoretical expectations [8]: under high-Na^+^ intake, large animals may rely more heavily on extrarenal storage mechanisms due to their relatively lower capacity to filter excess Na and relatively larger Na storage potential [8, 40]. The ability to store excess Na^+^ carries important implications for the biology of large mammals, including humans. Modern human diets are generally overloaded with Na^+^ [1, 7], and thus extrarenal Na^+^ storage is likely prevalent in a high number of people [5]. In contrast, many herbivorous mammals experience Na^+^ deficits [41, 42] and extrarenal Na^+^ storage my offer a safety buffer for times of deficiency. Indeed, a recent macroecological study highlighted that the density of African megaherbivores (animals > 1000 kg) appears to become constrained on dietary Na^+^ concentrations < 100 mg/kg [42]; for context, concentrations > 500 mg/kg are generally considered optimal for livestock [43, 44]. In these cases, if extrarenal Na^+^ storage occurs after periods of excess Na^+^ intake (e.g. from natural or articial salt licks [45]) it could be a mechanism that offers additional buffer for animals living in low-Na^+^ environments without only occasional access to concentrated Na^+^ resources (e.g., salt licks).

At present, however, the mobility of Na^+^ between connective tissue and other compartments of the body is not resolved, with earlier studies suggesting this was relatively fixed [46]. Notably, previous studies have usually relied on chronically different Na^+^ diets, rather than on a single pulse-dose, as undertaken in our cattle. Here, our results reveal that extrarenal Na^+^ storage is highly dynamic, with physiological responses changing within a few hours and reversible Na^+^ storage occurring within a few days. This aligns with evidence that diuretic treatments in humans can rapidly mobilise Na^+^ storage from skin tissues [47], while macrophages are linked to short-term changes in skin Na^+^ storage in rats [48].

To date, the majority of studies on Na^+^ homeostasis have been conducted with rats and humans [13, 16, 19, 24, 49]. While Na^+^ balance studies on smaller animals (e.g., rodents) may be logistically more feasible, examining physiological processes across body sizes, including animals larger than humans, may yield important insights for medical research, particularly related to hypertension [50]. Despite prolonged efforts to reduce salt consumption, hypertension remains a widespread medical condition throughout modern societies [1, 51]. Better understanding of the implications of high-Na^+^ diets across mammal body sizes (e.g., for cellular inflammation and disease [52]) can help modify Na^+^-related medical treatments in humans and public communication strategies [51].

### Future directions for measuring extrarenal Na^+^ storage in large mammals

We suggest that cattle—with their ease of handling and management—represent an attractive model organism to build on the insights we present here (Fig. 4). Future research could include undertaking parallel measurements of blood electrolyte concentrations, blood pressure, urine osmolarity, alternative excretion pathways (e.g., milk) and examination of Na^+^ and other ion concentrations within different organs across time series. We note that the use of a fistula as performed in the present study bypasses the animal’s taste buds, which may trigger different pshysiological responses [53, 54]. For future studies, however, it is not obligatory to use fistulated animals and it is experimentally feasible to administer any desired single pulse-dose of salt to cattle via one-time oesophageal tubing. In addition, cattle already designated for human consumption can easily be recruited as experimental animals that could be slaughtered at different time points after salt administration. NaCl is not a substance that legally or biologically prevents the use of experimental animals for meat, and the availability of large amounts of tissue makes sampling logistically feasible. In our study, we considered a response in water balance by examining the ratio of water intake and urine losses; the change over time, however, occurred in parallel to increasing food intakes and faecal output. Accurately tracking animal body mass throughout the experiment on consistent (restrictively fed) amounts of food may hold more accurate insights, while assessment of blood metabolites could be used to monitor if catabolism occurs following Na^+^ loading, as has been observed in small animal models [55].

Controlled experiments that administer doses scaled proportionally to body size or metabolic rate for different sized mammals (e.g., mice, rats, rabbits, sheep and cows) may help confirm if extrarenal Na^+^ storage scales allometrically across body sizes and which mechanisms drive deviations from these patterns [8]. We also only provided cattle with a single large pulse dose of NaCl (400 g per cow), yet comparative studies that administer various sized doses of Na^+^ may help elicit thresholds at which large mammals employ extrarenal Na^+^ storage. Ultimately, future experiments designed to examine the specific mechanisms of extrarenal Na^+^ storage across body sizes, such as the rate and magnitude with which different body tissues can store osmotically inactive Na^+^, may potentially hold novel insights for mammal physiology and medical research [8].

## Conclusion

Despite extrarenal Na^+^ storage being discussed as a potentially important component of total body Na^+^ balance for over a century [56, 57], there remain a number of outstanding questions regarding this mechanism [8, 13]. Here, we have shown data suggestive of extrarenal Na^+^ storage without concomitant losses of K^+^ in a large mammal species – cattle. This result excites a number of new research avenues of how Na^+^ regulation scales across body size, which may hold important insights for understanding Na^+^ balance and hyerptension in humans and mammals more broadly.

## Data Availability

Data associated with this paper is available at: https://doi.org/10.6084/m9.figshare.29064485.
